# On the Treatment of Burns and Scalds

**Published:** 1807-04

**Authors:** 


### *To the Editors of the Medical and Physical Journal.*

GENTLEMEN,

THE extraordinary dissonance of opinion existing among your Correspondents as to the method of cure to be adopted in Burns, and the equally extraordinary result of *success* from plans of treatment so diametrically opposite to each other, must create no small surprize in the mind of the unbiassed reader. As, at one period, I bestowed considerable attention on the subject in dispute, I shall feel obliged to you to give the subsequent observations, if you deem them worthy, a corner in your next Journal.

"In all controverted points," says Dr. Kinglake, with great great truth and propriety, "the ultimate appeal legitimately lies with facts." (p. 460) But when these *facts* directly controvert and clash with each other, how shall we be enabled to decide? It is evident however, that the contradictory relation of facts can never alter the nature or essence of *truth,* and that *truth* must lie somewhere; surely then, it cannot be considered either uncharitable or uncandid to suspect that *error* must exist either in the perception or detail of these facts. And here allow me to make a remark or two on this subject, utterly disclaiming however any personal allusion.—In the adduction of medical evidence there are two sources of error which have appeared to me peculiarly fruitful. The first is, *a deep-rooted prejudice in favour of any theory on the efficacy of any peculiar mode of treatment.* Under this species of *mental jaundice,* the Observer views every object tinged with *bile;* every thing has its *saffron* hue, more or less; and the defective color of one is atoned for by the more vivid appearance of another; till at length, by long looking through this deceptive medium, it becomes permanently substituted for natural perception. The second source, which is perhaps yet more productive of mischief, is, *a lively and luxuriant imagination uncontrolled by reason.* How far and how often this warps men from *truth* is daily instanced in "Voyages" and "Travels," of men who would start from an imputation of moral turpitude, and who yet contrive to give to the most trivial and insignificant incidents a zest and an interest, which a cool observer can seldom, by the most violent efforts, bring himself to believe they possessed. In medicine, by the friendly pliancy of one equivocal symptom, a stoical disregard of another, and a microscopical view of a third, which perhaps scarcely laid claim to existence, disease shall be metamorphosed into almost any shape or kind that is required. It should also be continually "had in remembrance," in submitting to public inspection cases that may tend to influence public opinion, that between absolute truth and falsehood there are a thousand gradations, a thousand shades of colouring; and that every deviation from the former is an approximation to the latter. There is another source of error, which is, perhaps, most frequently a ramification of the first above named, (but whether from choice or necessity affects not the position) and that is *viewing but one side of the question.* Now this I suspect to be immediately applicable to the matter in dispute. The Gentlemen who have in so decided and positive tive a manner condemned the plan of treatment recommended by Dr. Kentish, have never, it would appear, made trial of it in such a way as to enable them to appreciate its value. The *sum* of their evidence amounts to nothing more than this, "The cold-water treatment has in our hands been very successful; we are perfectly well satisfied with it; we disapprove of your opposite plan, because we think it *must* be hurtful, and besides, we do not like the theory." Let us see how a parity of this reasoning would look when applied to any other subject—suppose the intended introduction of the cow-pox. "The inoculated small-pox is very successful in our hands—comparatively few die of the disease naturally, and these only through the obstinacy of parents who will not allow them to be inoculated, and who would, probably, be still more averse to vaccination—we are perfectly well satisfied — and we disapprove of the cow-pox, because we think it *must* be an absurdity, and besides, you cannot produce any theory by which to explain to us the *modus operandi* of its prophylactic powers."—But "magna est veritas, et prevalebit." This has been amply verified in the progress of vaccination, and I am much mistaken if time do not also effect as much for the other.—The *negative* evidence of these Gentlemen then I aver to be incompetent to decide on the comparative merits of the two modes of treatment, and ought not by any means to weigh against the mass of respectable *positive* evidence which exists in its favour. Few men have had more extensive opportunities of observation than Dr. Kentish; and it was only from a thorough conviction of its superiority over the ordinary routine of practice, and this sanctioned by the superadded testimonies of many discriminating and unbiassed men, that he recommended his peculiar method to the attention of the medical world. Since then it has been progressively gaining ground, and is at this time followed in the Metropolis by some professional men of the first rank and talents.

As to the objections that have been adduced against its theory, most of them I think have originated in a defective acquaintance with ith. The terms "heating" treatment, "fiery" and "stimulant" applications, and other *inflammatory* epithets that have been used, are surely *incorrect* and *unphilosophical* when applied to the matter in question. As Dr. K. avowedly uses the terebinthinate application plication on the principle of its being a sedative,* that is I conceive, *a substance, the sum of whose exciting powers will on application to a sentient part produce an action inferior in degree to that previously existing in it*; *e. g.* if the excitability of a part be so far acted upon by external stimuli, as to produce excitement equal to 100 (supposing the natural standard 50), any substance whose powers would produce excitement equal only to 90, must certainly be *sedative,* as it bears reference to the parts it is to act upon, though in ordinary language it be termed *stimulant.* The terms *stimulant and sedative,* like *hot and cold, &c.* must always be considered as *relative* ones. Were two persons, one from a hot-house, the other from an ice-house, to enter a room of an intermediate temperature, to the feelings of the first it would seem *cold,* and to the other *warm.* The principle of Dr. K's practice is simply to lower the violent exciment produced by the contact of heat, *by degrees,* to the standard of healthy action. The *converse* of this doctrine is universally admitted, and acted upon with perfect success, in cases of limbs torpified by cold; as also in cases of accumulated excitability from the long-continued abstraction of the stimulus of food. In the one case *indirect debility* ensues, in the other *direct*; and it yet remains to be proved, that the rationale of practice in the instances ought to vary.

From Mr. Marson's communication in your last, I conclude he has not read Dr. Kentish's Essays, which it would certainly have been as well for him to have done before criticising his practice, and indirectly charging him with plagiarism. He would there have found himself anticipated in his quotations from Heister, &c.; and his *arch* remark concerning Dr. K's "coaxing" the medical world, as well as his recommendation of "*emollient and astringent practice,*" might have been spared.

I cannot better conclude these remarks than by *earnestly* recommending to the adoption of your numerous readers, the liberal and manly sentiments of Dr. Kinglake in the close

---

* That it *does* act as a sedative, the writer of this article would never wish for a more convincing proof than one which he witnessed about three years ago in a child, the daughter of a celebrated Engraver in London, who, from her clothes taking fire, was envelloped in flames till she was dreadfully burnt. The sp. terebinth. was profusedly applied, but one or two places having been overlooked, she eagerly and impatiently pointed to them, begging it might be applied there, which being done, her cries almost instantly ceased.

close of one of his Papers, (No. xci. p. 213), " Dr. Kentish's principle and practice shall receive from me all the investigation their importance deserves; and if they appear to be as well founded as Dr. K. conceives they are, I shall feel no difficulty in acceding to their high merit.

Every generous and unbiassed opponent should acquiesce in this— the most cordial admirer of Dr. Kentinsh's practice *ought* to wish for no more.

I am, &c.

C. N. W

*Newcastle, February* 28, 1807.

